# Prevalence of *Salmonella* spp. in slaughter pigs and carcasses in Irish abattoirs and their antimicrobial resistance

**DOI:** 10.1186/s13620-022-00211-y

**Published:** 2022-03-06

**Authors:** Annette Deane, Declan Murphy, Finola C. Leonard, William Byrne, Tracey Clegg, Gillian Madigan, Margaret Griffin, John Egan, Deirdre M. Prendergast

**Affiliations:** 1 Department of Agriculture, Food and the Marine, Backweston Complex, Celbridge, Co. Kildare Ireland; 2 School of Veterinary Medicine, Veterinary Science Centre, University College Dublin, Belfield, Dublin 4 Ireland

**Keywords:** *Salmonella*, Pigs, Ireland, Abattoirs, Carcass swabs, Serology, Caecal, Lymph nodes

## Abstract

**Background:**

*Salmonella* is an important zoonotic pathogen and is one of the main causes of foodborne outbreaks and infections in the European Union. Pigs are a significant reservoir and are frequently subclinical carriers of this organism. *Salmonella* can be shed in the faeces allowing infection to spread to other pigs, the environment, transport vehicles, lairages and other areas. Inadvertent spillage of gut contents during the slaughter process also leads to contamination. A pig *Salmonella* control programme has operated in Ireland since 2002 but many local surveys and an EUMS baseline survey in 2008 continued to indicate high levels of the organism in the pig sector. The objectives of this study were to generate updated information on the prevalence of *Salmonella* spp, in slaughter pigs and carcasses in Irish abattoirs. Five pigs from each of 164 herds were randomly sampled over a 14-week period during 2016. One sample from each of the five pigs of; caecal content, ileo-caecal lymph nodes and carcass swabs (pre-chill) were collected. The five caeca and lymph node samples from each herd were processed as one pool of caecal samples and one pool of lymph node samples, respectively, while the five carcass swabs were tested as individual samples. All isolates were characterised by serotyping and antimicrobial susceptibility.

**Results:**

In total, 235 *Salmonella* spp. were isolated from 820 individual carcass swabs, 164 pooled lymph nodes and 164 caecal contents. *Salmonella* spp. were isolated from 54.3% of the caecal contents and from 31.7% of the ileo-caecal lymph node sample pools. A total of 11.5% of carcass-swab samples yielded *Salmonella* spp. *S*. Typhimurium 4,[5],12:i:1,2 or its monophasic variant 4,[5],12:i:-: predominated among isolates from all positive samples; accounting for 73% of lymph nodes, 68% of caecal contents and 56% of carcass swab isolates. *S.* London and *S*. Derby were the next most common isolated serotypes.

**Conclusions:**

These results confirm continuing high levels of *Salmonella* in fattening pigs in Ireland although reductions in carcass contamination compared to previous surveys were noted. A high prevalence of *Salmonella* in lymph nodes suggests that it remains a significant problem pre slaughter and a challenge to abattoirs in adhering to process hygiene requirements. The high prevalence of monophasic *S*. Typhimurim 4,[5],12:i:-: is of serious concern.

Therefore, it is important to identify contributing factors in the dissemination of this pathogen in the pork industry in order to minimise the risk of human salmonellosis cases.

## Background

*Salmonella* is recognised as a major cause of food-borne illness in humans [1] with the poultry sector a major source. However, following the introduction of control programmes in EU Member States (EUMS), *Salmonella* levels in the poultry sector in EUMS have been greatly reduced as noted by EFSA and the ECDC and the focus of control in recent years has turned to the pig sector [2]. Pigs have been identified in a number of countries including Ireland as important carriers of *Salmonella* spp. and attribution studies show them to be an important source of human infection [2–5]. Pig meat is an important source of human infection [6] and it has been estimated that between 15 and 23% of all cases of human salmonellosis are related to the consumption of pork [2, 7, 8]. It should be noted that some *Salmonella* serotypes found in pigs are uncommonly associated with human infection whereas *S.* Typhimurium, and monophasic *S.* Typhimurium, important causes of human infection, account for about half of the isolates recovered from slaughter pigs across EUMS. Over 40% of pig *Salmonella* isolates that were serotyped and reported in 2019 were *S*. Typhimurium or monophasic *S*. Typhimurium [2, 9].

An EU-wide baseline study of *Salmonella* in slaughter pigs published in 2008 showed that 10.3% of slaughter pigs were *Salmonella*-positive, (based on testing of ileo-caecal lymph node samples), giving rise to concerns over risks to human health and the need to control and manage the disease and reduce that risk [10]. *Salmonella* prevalence in the pig sector varies across Europe; countries with long standing control programmes show lower infection levels in slaughter pigs [11]. A number of cost benefit studies have been undertaken to determine effective ways of introducing EU-wide controls [11] but to date there is no agreed strategy on control.

A legally defined *Salmonella* control programme has been operating in Ireland since 2002. Although the programme has been modified and refined on a number of occasions the basic principles have been retained. Herds are risk ranked on the basis of antibody tests on muscle juice samples [6] with individual abattoirs required to place additional controls on pigs processed from high-risk herds to minimise cross contamination of pigs and carcasses. Irish data from the EU baseline survey on the prevalence of *Salmonella* spp. in slaughter pigs in EUMS showed a *Salmonella* prevalence of 16.1% in ileo-caecal lymph nodes and 20% in carcass swabs, of which approximately 57% and 59% were *S*. Typhimurium, respectively [10]. A study of *Salmonella* in pigs at slaughter in Northern Ireland in 2002 showed the organism present in 31.4% of caecal contents and 40.0% of carcass swabs taken post-evisceration [12].

Serological test data show only limited changes to *Salmonella* antibody levels in Irish pigs since 2002 (DAFM unpublished). As the 2006–2007 baseline study was the last comprehensive study of *Salmonella* levels in Irish pigs, the current study was undertaken with the objective of updating baseline data of *Salmonella* in slaughter pig herds and carcasses, in order to obtain updated data to inform the future reviews of the National *Salmonella* Control Programme. Since completion of this survey, the results found have provided valuable updated information for a new “Pig Health Check” work programme initiated, since 2018, by Animal Health Ireland, which is a joint industry and government agency that works to improve animal health in farmed livestock in Ireland.

## Methods

### Bacteriology survey

The six main Irish pig abattoirs were recruited for the study and given targets for the number of herds to sample based on their annual throughput and number of suppliers. The sample collected was a convenience sample insofar as there were limitations both at the abattoir and in the laboratory governing the practicalities of sample collection and numbers that could be processed. Samples were collected from 164 of the total 300 commercial pig herds.

Five pigs from each of 164 herds (820 pigs in total) were randomly sampled by official veterinary inspectors at each abattoir over a 14-week period during 2016. From each of the five pigs, samples of caecal content, ileo-caecal lymph nodes and carcass swabs (pre-chilling) were collected. The five caeca and lymph node samples from each herd were processed as one pool of caecal samples and one pool of lymph node samples, respectively, while the five carcass swabs were tested as individual samples.

A standard protocol for the collection of samples was supplied to samplers with the logistics of collecting the samples in a suitable area away from the line being left to the discretion of the abattoir veterinary inspectors. Carcass swabs were collected pre-chilling using a commercial foam swab (Whirl–Pak®Speci-Sponge®Environmental Surface Sample Bags), approximately 100cm^2^ along the belly and extending down to the neck area on each side of the carcass (Chapter III of Regulation 2073/2005 (Microbiological Criteria). Samples were sent by overnight courier to the laboratory. Details of abattoir, the source of the pigs, the sampling date and time were entered onto a submission form, which accompanied the samples to the laboratory. Sampling commenced in early March 2016 and continued for a 14-week period.

### Laboratory methods

*Salmonella* isolation was carried out using a modification of the ISO 6579:2002 protocol [13]. In brief, all samples, 25 g caecal content, 25 g lymph nodes and each carcass swab were processed using an initial enrichment step at a 1:10 dilution in buffered peptone water (BPW) (Oxoid CM509). Lymph node samples were first washed in alcohol and then air dried to eliminate surface contamination. Selective enrichment in MRSV agar (Lab M) was followed by plating on both xylose lysine deoxycholate agar (XLD) (E&O Laboratories, Bonnybridge, Scotland) and brilliant green agar (BGA) (E&O Labs). *Salmonella* suspected isolates were plated onto chromogenic agar (E&O). Serotyping was carried out as per the White-Kauffmann-Le Minor classification scheme [14].

### DNA extraction and PCR confirmation of monophasic variant 4,[5],12:i:-:

PCR was performed on the 97 isolates for which conventional serotyping showed an incomplete antigenic formula that shared antigens with the *S*. Typhimurium formula 4,[5],12:i:1,2 i.e., for which formulas 4,[5],12:i:-: were obtained. Isolates were cultured overnight on Nutrient Agar (Lab M Ltd.,Lancashire, UK) at 37 °C. One isolated colony was suspended in 100 µl InstaGene™ Matrix (BioRad laboratories, USA) and DNA was extracted as per manufacturer’s instructions. A multiplex real-time PCR method for the identification and differentiation of *S.* Typhimurium and monophasic 4,[5],12:i:- was carried out using the method and primers as described by Prendergast et al. 2013 [15].

*S*. Typhimurium LT2 ATCC 29,946 was used as a positive control and *Escherichia coli* NCTC 9001 as a negative control.

### Antimicrobial susceptibility testing

Antimicrobial susceptibility testing was conducted on two hundred and thirty-five isolates using the Sensititre broth microdilution method for *Salmonella*, EUVSEC (Sensititre, TREK Diagnostic Systems Inc., Sussex, England) and in accordance with the Clinical and Laboratory Standards Institute (CLSI) methods [16]. Custom-made panels of 14 antimicrobial drugs at specified concentrations were configured in 96-well microtiter plates. The panel of antimicrobials and the cut-off values (mg/l) were in agreement with the EU commission implementing Decision of 12 November 2013 on the monitoring and reporting of antimicrobial resistance in zoonotic and commensal bacteria [17], as follows: Ampicillin (AMP) (1–64 mg/l), Cefotaxime (CTX) (0.25–4 mg/l), Ceftazidime (CAZ) (0.5–8 mg/l), Meropenem (MER) (0.03–16 mg/l), Nalidixic acid (NAL) (4–128 mg/l), Ciprofloxacin (CIP) (0.015–8 mg/l), Tetracycline (TET) (2–64 mg/l), Colistin (COL) (1–16 mg/l), Gentamicin (GEN) (0.5–32 mg/l), Trimethoprim (TMP) (0,25–32 mg/l), Sulfamethoxazole (SMX) (8–1024 mg/l), Chloramphenicol (CHL) (8–128 mg/l), Azithromycin (AZI) (2–64 mg/l), Tigecycline (TIG) (0.25–8 mg/l). The wells of the microtiter plates were manually read for bacterial growth using the Sensititre Vizion System (TREK disgnostic Systems). *E. coli* ATCC 25,922 was used as the quality control strain for this assay. The AMR result was designated as “Fully Susceptible” or by indicating the abbreviation of the antimicrobial to which the strain was resistant [18].

### Data analysis

All data were entered into a Microsoft Excel (Microsoft Corporation) spread sheet with visual checks for accuracy made at the point of entry, and again at the end of the survey. After validation, data were transferred to, and all statistical analyses carried out using SAS version 9.4. Serology data for each herd sampled was taken from the national monitoring programme database and merged with the bacteriology data.

### Monthly serological prevalence

The average serological prevalence per month was calculated by summing the total number of tests conducted and total number of positive samples within a month over the years 2010 to 2016. Estimates for January to June were based on 7 years of data and those for July to December were based on 6 years of data.

### Herds with the highest and lowest serological prevalence

Serology tests carried out from July 2015 to June 2016 were used to rank herds according to the serology prevalence within the herd over this time period. Herds in the top and bottom 10% of herds, based on serology prevalence, were compared for the bacteriology results on lymph nodes, caeca and carcass swabs. A chi-squared test was used to compare herds in the top and bottom groups, except when the expected number in a cell was < 5 in which case a Fisher’s exact test was used.

### Probability of a positive carcass swab

A logistic regression model was developed to model the probability of a herd having a positive carcass swab. The results of the following independent variables were tested within the model: caecal contents, lymph nodes, caecal contents /lymph nodes, the processing plant and the herd serological result. For the serological result the following time periods were considered for inclusion within the model: Jan to July 2016; June 2015 to July 2016 and Jan 2010 to July 2016. Initially each independent variable was tested within a univariable model. The best fitting time period to use for serological results within the multivariable model was determined by comparing the AIC (lowest value gave the better fit) of each time variable in the respective univariable model. Similarly, the choice of whether to include caecal and lymph results separately or combined was determined by the AIC of the univariable models. A backward selection procedure was used to eliminate terms from the model based on a likelihood ratio test.

## Results

*Salmonella* spp. were isolated from the pooled caecal contents of 91 (55.5%, 95% confidence interval (CI) 46.3–62.0) of the 164 herds and from the ileo-caecal lymph nodes in 52 herds (31.7%, 95% confidence interval (CI) 24.8–39.5). A total of 94 (11.5%, 95% confidence interval (CI) 9.4–13.9) of the 820 carcass-swab samples yielded *Salmonella* spp. (Table 1).

**Table 1:** Total number of animals sampled, and type and number of samples positive for *Salmonella* in 6 abattoirs

			**Number of Positive**			**Number of Positive**	
			**Pooled Samples (%)**^**a**^			**Carcass Swabs (%)**^**b**^	
**Abattoir**	**Herds**	**Animals**	**Caeca**	**Lymph nodes**	**Caeca/Lymph nodes**	**Positive herds**	**Positive pigs**
A	22	110	11 (50)	5 (22.7)	11 (50)	6 (27.3)	6 (5.5)
B	30	150	17 (56.7)	10 (33.3)	17 (56.7)	13 (43.3)	25 (16.7)
C	40	200	19 (47.5)	8 (20)	20 (50)	7 (17.5)	10 (5)
D	26	130	14 (53.8)	5 (19.2)	15 (57.7)	4 (15.4)	5 (3.8)
E	25	125	19 (76)	15 (60)	19 (76)	8 (32)	10 (8)
F	21	105	11 (52.4)	9 (42.9)	12 (57.1)	12 (57.1)	38 (36.2)
Total	164	820	91 (55.5)	52 (31.7)	97 (59.1)	50 (30.5)	94 (11.5)

^a^Pools of 5 samples were tested in the case of caecal and lymph node samples and thus each pooled sample represents the result for one herd

^b^Carcass swabs were tested individually; 5 samples were collected per herd

The distribution of *Salmonella* serotypes found and the abattoir, from which the positive samples were collected, is shown in Table 2. The most dominant serotype from each sample type was mST. Among the 52 *Salmonella* isolated from lymph nodes, 38 (73%) of these were either serotyped as *S*. Typhimurium i.e., 4,[5],12:i:1,2 or as its monophasic variant, mST 4,[5],12:i:-: with the remaining serotypes identified as *S*. London (4), *S*. Kimuenza (2), *S*. Derby (2), *S.* Infantis (1), *S*. Goldcoast (1), *S*. Enteritidis (1), *S*. Mbandaka (1) and *S*. Unnamed (2). The two *S*. Unnamed isolates from lymph nodes had antigenic formulas 4:-:2 and 6,7:-:5. Among the 91 *Salmonella* isolated from caecal contents, 61 (67%) were either serotyped as *S*. Typhimurium or as mST with the remaining serotypes identified as *S*. London (9), *S*. Derby (6), *S*. Kimuenza (4), *S*. Infantis (2), S. Anatum (2), *S*. Goldcoast (1), *S*. Enteritidis (1), *S*. Manhattan (1), *S*. Putten (1), *S.* Cubana (1) and *S.* Unnamed (2). The two unnamed isolates had antigenic formulas 4:-:2 and 6,8:-:5 (Table 2).

**Table 2:** Main *Salmonella* serotypes isolated from *pooled caecal content and lymph node samples and individual carcass swabs collected in each of 6 abattoirs

			Number Positive (% of isolates from same sample type and same abattoir)								
Sample Type	Abattoir	Total isolates per abattoir	Mono ST	ST		*S*. Kimuenza	*S*. London	*S*. Derby	*S*. Infantis	*S*. Anatum	Other
*Lymph Node	A	*n* = 5	2 (4)		1(2)	-	-	-	-	-	2(4)
	B	*n* = 10	5(10)		3(6)	-	-	-	1(2)	-	1(2)
	C	*n* = 8	4(8)		3(6)	-	-	-	-	-	1(2)
	D	*n* = 5	1(2)		4(8)	-	-	-	-	-	-
	E	*n* = 15	6(12)		5(10)	-	4(8)	-	-	-	-
	F	*n* = 9	3(6)		1(2)	2(4)	-	2(4)	-	-	1(2)
*Caecal Contents	A	*n* = 11	4(4)		4(4)	-	1(1)	1(1)	-	-	1(1)
	B	*n* = 17	7(8)		6(7)	1(1)	-	-	1(1)	-	2(2)
	C	*n* = 19	11(12)		3(3)	-	-	1(1)	-	2(2)	2(2)
	D	*n* = 14	9(10)		2(2)	-	1(1)	1(1)	1(1)	-	-
	E	*n* = 19	9(10)		1(1)	-	7(8)	1(1)	-	-	1(1)
	F	*n* = 11	3(3)		2(2)	3(3)	-	2(2)	-	-	1(1)
Carcass Swabs	A	*n* = 6	6(6)		-	-	-	-	-	-	-
	B	*n* = 25	17(18)		4(4)	1(1)	-	1(1)	-	-	2(2)
	C	*n* = 8	5(5)		-	-	-	1(1)	-	-	2(2)
	D	*n* = 5	-		5(5)	-	-	-	-	-	-
	E	*n* = 10	3(3)		3(3)	-	1(1)	1(1)	-	-	2(2)
	F	*n* = 38	7(7)		3(3)	25(27)	-	3(3)	-	-	-

Ninety-two *Salmonella* were isolated from carcass swabs and among these, 53 (57%) were serotyped as *S*. Typhimurium or as mST with the remaining serotypes identified as *S*. Kimuenza (26), *S*. London (1), *S.* Derby (6), *S*. Gloucester (1), *S*. Rissen (1) and *S*. Unnamed (4). The four *S*. Unnamed isolates had antigenic formulas 4:-:2, 4:l,v:-:, 13:-:-: and 13:f,g:-: (Table 2).

### Herds with the highest and lowest serological prevalence

Herds in the bottom 10% had an average serological prevalence of < 1.3% while those in the top 10% had an average prevalence of > 37.9%. There were no significant differences in any of the bacteriological results between herds in the top and bottom 10% serological prevalence groups (data not shown).

### Probability of a positive carcass swab

**Fig. 1:**
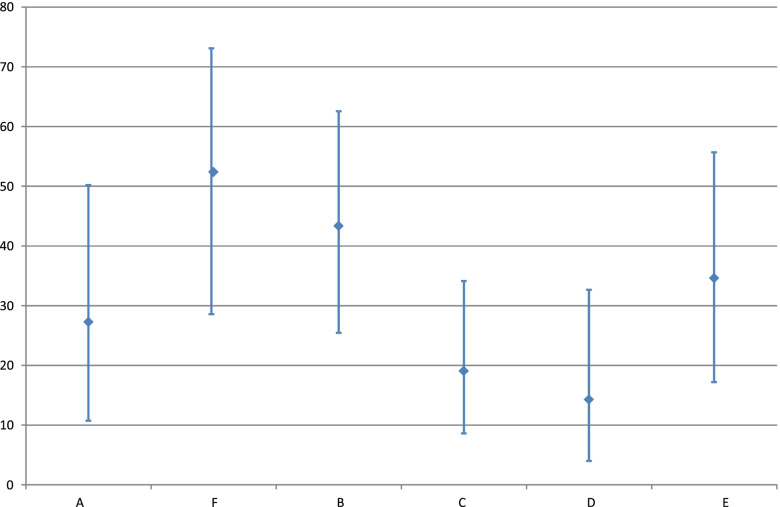
Percentage of herds with a positive carcass swab by processing plant

Figure 1 shows the proportion of herds from each of the 6 abattoirs with a positive carcass swab along with 95% confidence intervals. All of the confidence intervals overlap; Abattoir F had the highest proportion of positive carcass swabs and Abattoir D had the lowest.

The final logistic regression model developed to model the probability of a herd having a positive carcass swab contained just the lymph node variable; all other variables were not significant and were excluded from the final model. The odds of a herd having a positive carcass swab were 2.69 (95% CI 1.35–5.39) times higher when the herd had a positive lymph node result compared to herds with a negative lymph node result (*P* = 0.005).

### Antimicrobial resistance

The antimicrobial resistance profiles of the two hundred and thirty-five isolates tested are shown in Table 3. Among the 102 mST 4 [5], 12:i:-, one isolate was resistant to 6 antimicrobials (AMP CHL GEN TET TIG TMP), 34 were resistant to 5 antimicrobials (AMP CHL GEN TET TMP), 22 were resistant to different combinations of 4 antimicrobials and 32 isolates displayed AMR profiles of AMP TET. In general, the AMR profiles of the 50 *S*. Typhimurium isolates differed from those of mST. The predominant pattern in mST isolates was resistance to AMP CHL GEN TET TMP whereas the most common pattern in the ST isolates was resistance to AMP CHL TET.

**Table 3:** Antimicrobial resistance profiles of *Salmonella* (*n* = 235) from lymph nodes, caecal samples and carcass swabs

AMR Profile	Monophasic variant	*S.* Typhimurium	*S*. Derby	Other serovars	Total
AMP CHL CIP GEN TET TIG TMP				1	1
AMP CHL GEN TET TIG TMP	1				1
AMP CHL GEN TET TMP	34	3		2	39
CHL CIP TET TIG TMP				1	1
AMP CHL TET TIG TMP		2		1	3
CHL TET TIG TMP		1			1
AMP GEN TET TMP	2	1			3
AMP CHL TET TMP	4	4			8
AMP CHL CIP TET	1				1
AMP GEN TET	15	1		1	17
AMP CHL TET		14		1	15
AMP TET TMP	2	1		1	4
CIP NAL				2	2
TET TMP			8		8
CHL TET		1			1
AMP TET	32			3	35
TMP		2			2
TET	2	5	3	2	12
AMP	5	8			13
Fully Susceptible	4	7	3	54	68
**Total number of isolates**	**102**	**50**	**14**	**69**	**235**

The 69 isolates of serotypes other than *S*. Typhimurium, mST, and *S*. Derby were grouped together as the majority of these were fully susceptible, i.e., 54 isolates were fully susceptible. Within this group of serotypes, eight were multi drug resistant displaying profiles of AMP CHL CIP GEN TET TIG TMP (1), AMP CHL GEN TET TMP (2), CHL CIP TET TIG TMP (1), AMP CHL TET TIG TMP (1), AMP GEN TET (1), AMP CHL TET (1) and AMP TET TMP (1); the two *S*. Goldcoast isolates showed AMR profiles of AMP CHL CIP GEN TET TIG TMP and CHL CIP TET TIG TMP. The three untypable 4:-:2: isolates were multi drug resistant with AMR profiles of AMP CHL GEN TET TMP, AMP CHL TET and AMP CHL TET TIG TMP. One untypable 6,8:-:5: isolate displayed an AMR profile of AMP, CHL, GEN, TET, TMP and one *S*. Gloucester isolate had an AMR profile of AMP, GEN, TET. The remaining 7 isolates consisting of two *S*. Anatum, one *S.* Rissen, one *S*. Kimuenza and three untypable isolates of antigenic formula 13:f,g:-, 13:-:-: and 4:l,v:-: displayed AMR profiles of CIP NAL, TET, AMP TET, AMP TET, AMP TET and TET, respectively.

## Discussion

*Salmonella* spp. were isolated from 91 (55.5%) of pooled caecal contents and 52 (31.7%) of pooled ileo-caecal lymph nodes from 164 herds sampled, which represent about half of the commercial pig fattening herds in Ireland. The much lower rate of detection, 11.5% (94 of 820), in carcass-swab samples, is indicative of improvements in the effectiveness of process hygiene controls, despite the high levels of caecal and lymph node positive samples.

*Salmonella* contamination of carcasses is a key parameter used to measure the effectiveness of process hygiene criteria of the abattoirs. While the rate of *Salmonella* detected on carcasses in the present study was higher than the EU average of 10.3% which was reported in the baseline study [10], nevertheless, the reduction in *Salmonella* contamination of carcasses (to 11.5%) in this study compared to that of the baseline study (20%) is a positive development. This reduction was achieved despite a possibly increased *Salmonella* prevalence in the fattening pigs where carriage rates, as reflected by ileo-caecal lymph node *Salmonella* prevalence increased from 16.1% during the baseline survey to 31.7% in this study. A further reduction in the proportion of carcasses from which *Salmonella* spp. were detected to; 4.7%, 4.2% and 5.9% in the years 2018, 2019 and 2020, respectively, has been reported from results of official testing in accordance with Commission Regulation (EC) No. 218/2014 [2, 9, 19]. However, the results between the baseline and this present study are not directly comparable since the baseline survey refers to the prevalence from individual pigs whereas this study refers to prevalence in pooled samples from 5 pigs.

Isolation of *Salmonella* serotypes from the ileo-caecal lymph nodes is frequently considered to reflect *Salmonella* prevalence on farm; however short-term exposure in the lairage can also lead to lymph node positivity [20, 21]. The high prevalence in lymph nodes suggests that *Salmonella* remains a significant (and possibly an increasing) problem at farm level and in the lairage area and a challenge to abattoirs in adhering to process hygiene requirements.

Although many studies on *Salmonella* in pigs in EUMS have been published, the baseline study [10] provided a uniform approach in which methods of sampling and testing were standardised across all participating countries. Factors such as differences in the nature of the swabs used, the areas swabbed and in many cases the point at which the swabs were taken, must be considered, in any comparison of the carcass swab results in the present study to those found in other studies. All the sampling done in our study was undertaken under official veterinary supervision to ensure consistency of approach between different abattoirs. The results of the present study are not unlike those found in Northern Ireland and England although culture methods varied. During 2002, *Salmonella* spp. was isolated from 31.4% of caecal contents and 40.0% of pre chill carcass swabs in Northern Ireland [12]. The UK baseline study identified *Salmonella* in 21.2% of ileo-caecal lymph nodes and 13.5% of carcasses [1]. An earlier UK survey during 2000 [22] reported carriage of *Salmonella* in 23.0% of caecal contents. Further studies found *Salmonella* spp. in 23.4% of pigs sampled during 2003 [23]. A more recent study conducted in the UK during 2013 [24] reported a *Salmonella* prevalence of 30.5% in caecal contents and 9.6% of carcass swabs, which are very similar to the results of this present study (caecal contents = 31.7%; carcass swabs = 11.5%). In addition, process hygiene criterion monitoring data on *Salmonella* from pig carcasses for 2019, reported a prevalence of 3.15%, compared to 6.7% of *Salmonella*-positive samples from carcasses during 2006 / 2007 [10]. Other studies provide further information on the prevalence of *Salmonella* in pigs in the Republic of Ireland. A study during 2001 identified *Salmonella* in the caecum samples of 37% low risk herds and 66.6% higher risk herds [25]. Another study conducted during 2007, reported a *Salmonella* prevalence of 14.8% and 11.7% of lymph nodes and caecal contents respectively, and 10.2%, 3.9%, 1.8%, and 7.2% of pre-wash, post wash, post chill and belly-strip carcass swabs, respectively [26]. Bolton et al., 2013 [27] investigated the prevalence of *Salmonella* in pigs from birth to carcass and reported a prevalence of 27.5% and 5% of throat/rectal and carcass samples positive for *Salmonella*, respectively.

The results of this study shows that *S*. Typhimurium and mST predominated in all samples, accounting for 65% of all isolates, 67% of caecal content isolates, 73% of lymph node isolates and 57% of carcass swab isolates. The predominance of *S*. Typhimurium and mST reported among porcine *Salmonella* isolates here is similar to that reported in the EU baseline survey and other surveys in Ireland and elsewhere. However, while the predominance of *S.* Typhimurium and mST, collectively, has continued it is noteworthy that the relative proportion of isolates that are mST (*n* = 102) relative to *S*. Typhimurium (*n *= 50) has increased in recent years and mST is now widely seen across Europe as the more prevalent serotype in pigs and pigmeat [28–31]. Overall, the prevalence of mST relative to samples positive was 43.4% in this present study compared to 21.3% for *S*. Typhimurium which is very similar to that (39%) reported by Mueller-Doblies et al. [28] during 2014. It is also noteworthy that mST was more predominant than ST in the present study from each porcine sample type with 40.4%, 47.3% and 41.3% mST identified in lymph nodes, caecal contents and carcass swabs respectively compared to 32.7%, 19.8% and 16.3% positive for *S*. Typhimurium. During 2017, mST were among the most commonly reported serotypes isolated from pigs in the EU. Pigs and pig meat accounted for, 167 (37.4%) and 129 (22%) of mST isolates respectively. These results confirm that pigs are the main reservoir for mST and the results of the study reported here support this conclusion.

The levels of antimicrobial resistance detected, with high levels of resistance to ampicillin, chloramphenicol, streptomycin, sulphonamides, tetracycline and trimethoprim, in *S*. Typhimurium and mST are comparable to other studies [22, 32]. For all serotypes, resistance to nalidixic acid and ciprofloxacin (1.7%), was low compared to other studies where resistance in all *Salmonella* serotypes to nalidixic acid alone was reported to be 4.1% in GB and 6.5% Spain [22, 32]. In both of these previous studies nalidixic acid resistance was common in *S.* Typhimurium. A study conducted in the Republic of Ireland by Bolton et al. 2013 [27] reported a high level of resistance of *S*. Typhimurium to streptomycin, sulfonamides, tetracyclines and trimethoprim that are commonly used individually or in combination in veterinary medicine [33]. In this study, 94 (40%) of isolates were resistant to at least three or more antimicrobials, 41 (17.4%) of isolates were fully susceptible and 27 (11.5%) of isolates were resistant to only one antimicrobial. Among the 94 isolates resistant to three or more antimicrobials, 59 (62.7%) were mST, 27 (28.7%) were *S*. Typhimurium and 8 (8.5%) were a combination of other serotypes. These results provide more evidence that mST and *S*. Typhimurium have developed a high level of resistance in particular to older antibiotics which have been used over a long period of time in the pig industry. Antimicrobials, to which the highest frequencies of resistance detected in *E. coli* isolates from pigs have been reported previously to be tetracycline, trimethoprim/sulphamethoxazole and streptomycin, with the highest levels of resistance in weaned pigs [34]. The high level of multidrug resistance observed, particularly in mST is consistent with other research reporting this variant to be a host of multiple drug resistance [35].

Reducing *Salmonella* contamination of pig meat products is dependent on effective control on the farm and in the abattoir. *S.* Typhimurium is endemic on a significant proportion of Irish pig farms and further research is required to determine factors which have created a favourable environment for the establishment and persistence of this organism. However, progress in reducing *Salmonella* contamination of pork carcasses could likely be made in the short term by focussing on further improvements during the slaughter process [26].

## Conclusions

The results of this study indicate a decrease in *Salmonella* contamination of carcasses in Ireland since the EU Baseline study in 2008, most likely due to improvements in hygiene control measures during processing. However, as *Salmonella* spp. isolates were recovered from a higher percentage of ileo-caecal lymph nodes, there remain a significant and possibly growing prevalence of *Salmonella* spp in fattening pigs. The high level of multidrug resistance in mST serotypes is a cause for concern. These results coupled with the results of the on-going serological monitoring of fattening pigs indicate that the control programme may be having little impact at farm level. The prevalence of *Salmonella* spp. in pigs and the difficulty in its control are well recognised and attempts for coordinated EUMS controls have not been feasible. The continuing high prevalence of *Salmonella* in fattening pigs should focus attention on the need for further controls at farm level and a re-examination of the current control programme.

## Data Availability

The datasets used and/or analysed during the current study are available from the corresponding author on reasonable request.
